# Supplementary material for: Broad misappropriation of developmental splicing profile by cancer in multiple organs
Source: Nat Commun. 2022 Dec 12;13:7664. doi: 10.1038/s41467-022-35322-1 (PMC9744839; doi:10.1038/s41467-022-35322-1)
Supplement: Supplementary file 1 — Supplementary Information [file 41467_2022_35322_MOESM1_ESM.pdf]

Supplementary Figure 1

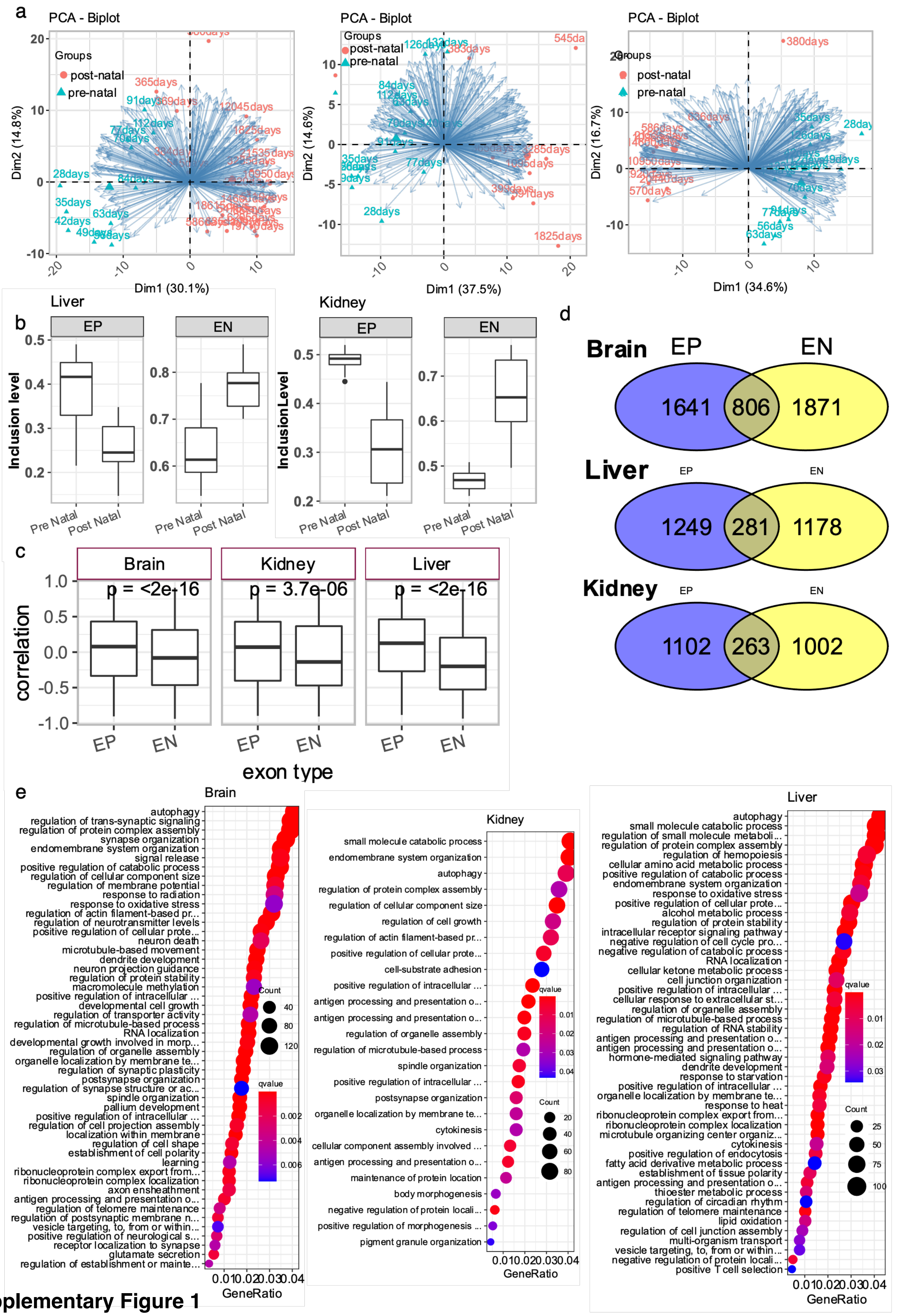

Supplementary Figure 1

a Biplot from the principal component analysis of the KEGG pathway scores during the developmental timeline of three tissues. Text colors in the figure denote the pre- and post-natal stages of development **b** Boxplots showing the differential inclusion of embryonic positive (EP) and embryonic negative (EN) events during pre-natal (n = 14 for liver and kidney) and post-natal (n = 10 for liver and n = 8 for kidney) stages of development for kidney and liver (related to figure 1E). **c** Distribution of Pearson's correlation coefficient between the inclusion level of an EP (n = 3069 for brain, n = 1588 for kidney and n = 1724 for

liver) or EN (n = 3477 for brain, n = 1449 for kidney, and n = 1648 for liver) exons and the expression and the expression level of their host gene across the developmental data of brain, kidney, and liver. Two-sided p-values from Wilcoxon's test are shown. **d** Venn diagram for the overlap between the list of host genes for EP and EN events in three tissues, emphasizing that same gene might contain EP and EN events. **e** Dot plots for the GO terms (biological processes) enrichment among the host genes of EP and EN events in three tissues. Dots are colored based on FDR corrected one-sided p-value from Fisher's test (labelled as q-value) as implemented in clusterProfiler package in R and sized based on the number of genes in each functional category. In boxplots (**b, c)**, the horizonal line in the middle is the median value and the lower and upper edges of the boxes correspond to the 25th and 75th percentiles. Extending vertically upwards/downwards of the boxes are the lines showing 1.5 times the interquartile range (i.e., distance between 25th and 75th percentile). Dots are the outliers. Source data for these figures are provided as a Source Data file.

Supplementary Figure 2

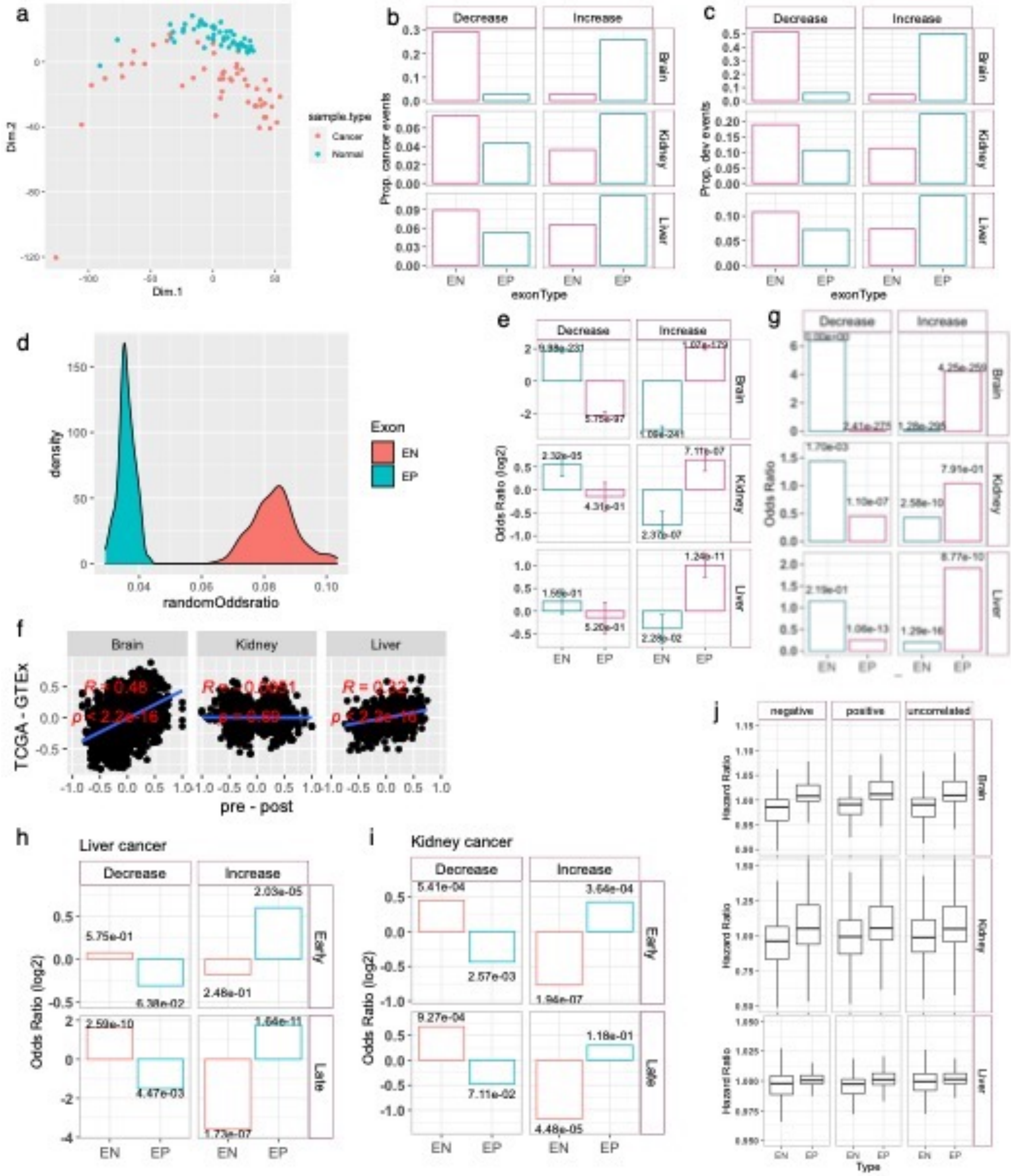

Supplementary Figure 2

a Scatter plot of liver hepatocellular carcinoma (LIHC) samples along the first two PCs based on the global inclusion level (PSI value) of exon skip events. **b, c** The proportion of cancer-specific increased and decreased events (based on TCGA vs. GTEx comparison; Methods) among the EP and EN events (**b**) and the proportion of EP and EN events among the cancer-specific events (**c**). **d** Null distribution for the odds ratio of enrichment (from Fisher's test) of randomly sampled exons among the events frequently increased and decreased in cancer. These random exons were sampled to have low inclusion (PSI < 0.30) and high inclusion (PSI > 0.70) for EP and EN events, respectively, in GTEx tissues, ensuring that the enrichment of EP and EN events in cancer is not resulting from their baseline inclusion level in adult tissues. **e** Bar plots showing the odds ratio and 95% confidence intervals (whiskers) calculated using

Fisher's test for the enrichment of EP and EN events among the cancer-specific events after removing all the EP events which had near zero inclusion in normal GTEx samples (i.e., PSI < 0.05 in > 80% of the samples) Shown are the FDR adjusted two-sided p-values from Fisher's exact test. **f** Scatter plot for the "pre-natal – post natal" ΔPSI of splicing events (x-axis) against their "TCGA – GTEx" ΔPSI in cancer. Blue lines and shaded grey areas respectively depict the best fitting lines and 95% confidence intervals based on linear regression, Pearson's correlation coefficients and two-sided p-values are shown in the plots. **g** Bar plots showing the odds ratio of enrichment/depletion for the EP and EN events among the cancer-specific events. The cancer-specific events were identified using a Wilcoxon's test to assess the differential inclusion (|ΔPSI| > 0.2 & FDR < 0.1) of each exon in TCGA relative to GTEx. The FDR adjusted two-sided p-values of enrichment (odds ratio) are shown. At a stringent FDR threshold of odds ratio (FDR <= 0.01), a total of 10 out of 12 comparisons show the expected trend, i.e., the enrichment of EP/EN events among events increased/decreased in cancers and depletion of EP/EN events among the events decreased/increased in cancers. At relaxed FDR threshold of 0.25, 11/12 comparisons were significant. **h, i** Odds ratio for the enrichment of EP and EN events among frequently increased and decreased events in the early and late-stage cancers, showing the greater reversion to embryonic splicing in advanced stage cancers. In G-I, Odds ratio was calculated using the Fisher's exact test and numbers near each bar are the FDR adjusted two-sided p-values of the Fisher's test. **J** Hazard ratio for EP and EN exons separated into three subclasses; namely negative (n = 1309 for Brain EN and 853 for Brain EP, n = 480 for Kidney EN and n = 401 for Kidney EP), positive (n = 917 for Brain EN and n = 1098 for Brain EP, n = 331 for Kidney EN and n = 439 for Kidney EP), and uncorrelated (n = 1230 for Brain EN and n = 1098 for Brain EP, n = 529 for Kidney EN and n = 576 for Kidney EP) depending on the correlation of their inclusion level with the expression level of host gene, showing that hazard ratios of EP and EN exons is independent of the expression of their host gene. In boxplots, the horizonal line in the middle is the median value and the lower and upper edges of the boxes correspond to the 25th and 75th percentiles. Extending vertically upwards/downwards of the boxes are the lines showing 1.5 times the interquartile range (i.e., distance between 25th and 75th percentile). Dots are the outliers. Source data for these figures are provided as a Source Data file.

Supplementary Figure 3

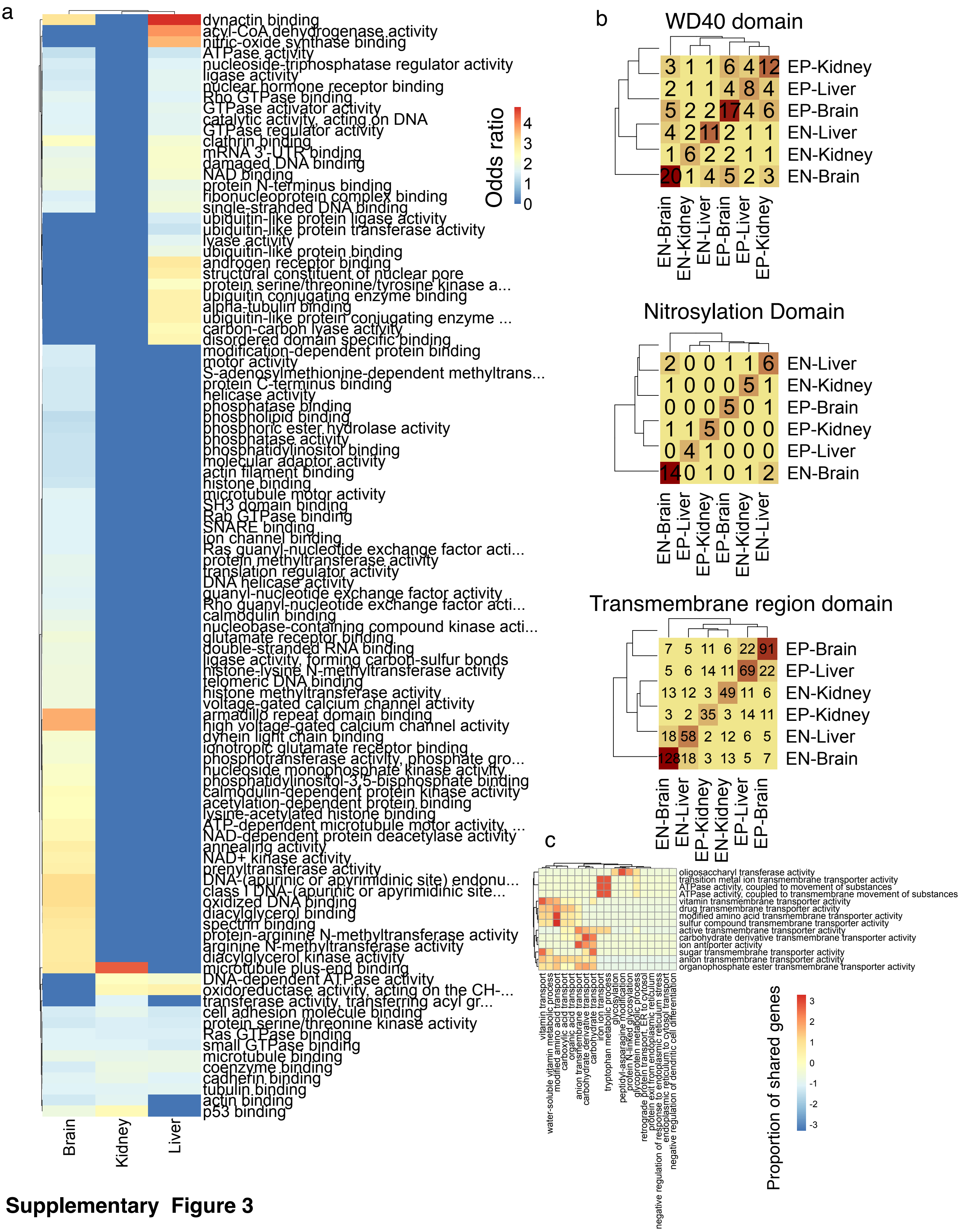

Supplementary Figure 3

a Heatmap for the GO term (molecular function) enrichment analysis of host genes of EP and EN events in three tissues. The colors in heat cell correspond to –log10 of FDR corrected one-sided p-value of enrichment from Fisher's test as implemented in clusterProfiler library in R. **b** Heatmap for the overlap among the gene sets enriched for WD40, nitrosylation and transmembrane-region domains across EP and EN events in three tissues. For each domain (i.e., the three plots), the numbers along the diagonal indicate the number of genes having that specific domain and off-diagonal entries show the number of common host genes of EP/EN events across tissues. This plot emphasizes that for a given domain, the observed enrichment of these three domains (Fig. 3a) is driven by different set of genes. The colors in the heatmap codes the value written in each cell. **c** Correspondence between molecular functions and biological processes for transmembrane domains in liver EP events. Source data for these figures are provided as a Source Data file.

Supplementary Figure 4

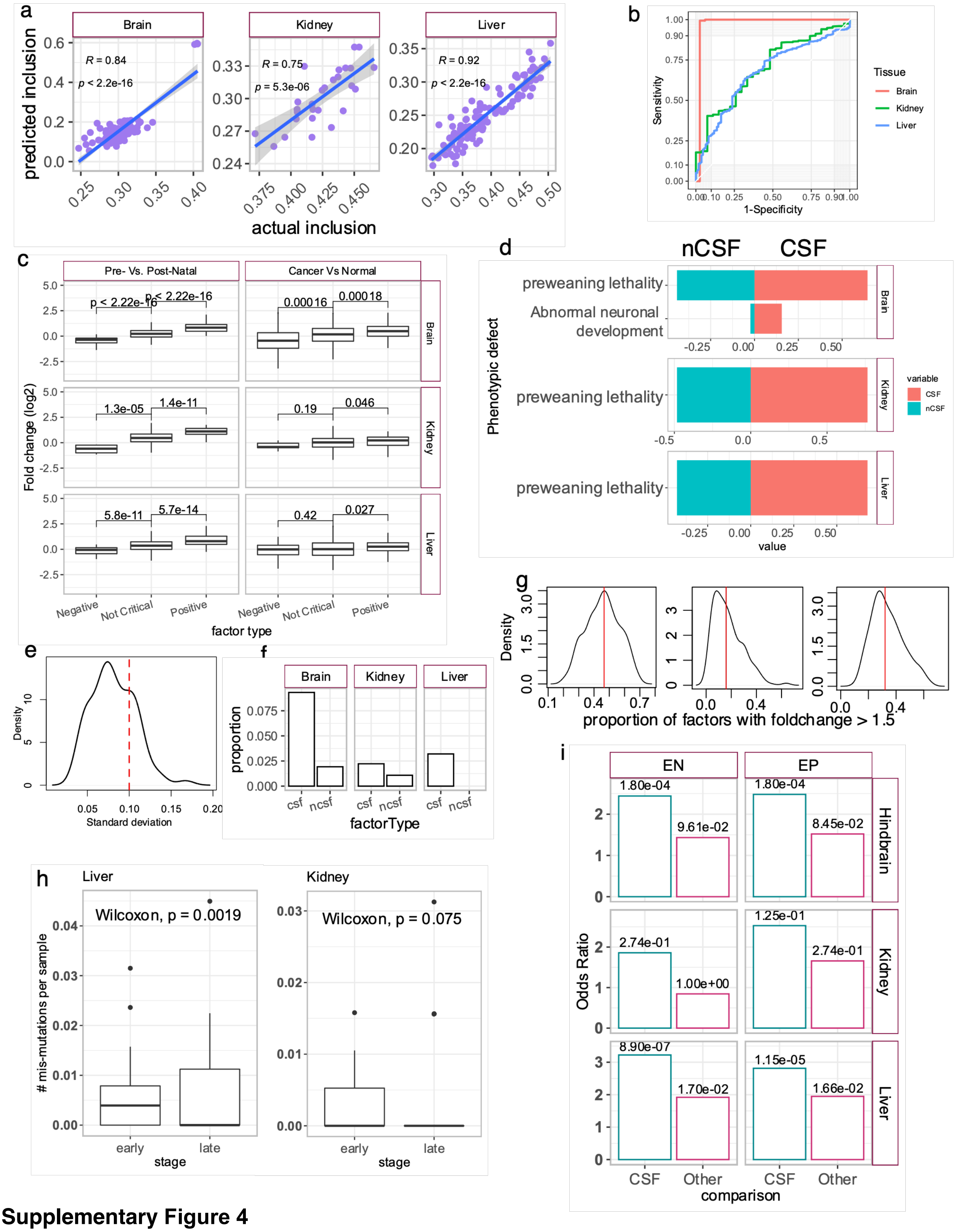

Supplementary Figure 4

a Scatterplot between the actual and predicted median inclusion level of EP splicing for brain, kidney, and liver in the GTEx cohort. Pearson's correlation coefficients and p-values are shown in the plots. Blue lines and shaded grey areas respectively depict the best fitting lines and 95% confidence intervals based on linear regression between actual and predicted median inclusion of EP events. **b** ROC curve for the classification of TCGA and GTEx samples into normal and tumor category using the predicted median inclusion level of the EP events based on PLSR model. Shown are the area under the curve (AUC) values. **c** Boxplots showing the distribution of fold change in the gene expression for splicing factors with

non-significant (FDR > 0.05, not-critical group in the plot, (n = 241 for brain, n = 387 for kidney, and n = 200 for liver), and significantly (FDR < 0.05) positive (Positive group in the plot, n = 119 for brain, n = 45 for kidney, and n = 167 for liver), negative (Negative group in the plot, n = 81 for brain, n = 9 for kidney, and n = 74 for liver) regression coefficients. **d** Phenotypic consequences of deletion/knockdown of critical (CSF) and non-critical (nCSFs) splicing factor orthologs in mice. Bar plots show the fraction of CSFs (red) and nCSFs (blue) that results in developmental phenotypic defects (shown next to bar plots) in mice. The deletion of brain CSFs results in abnormal nervous development. CSFs from all three tissues are much more likely to result in pre-weaning lethality as compared to CSFs. Phenotypes shown here had an enrichment (OR > 1) of CSFs as compared to nCSFs at a two-sided p- value threshold of 0.1 and FDR of 0.30 from Fisher's exact test. For this analysis, we complied the list of phenotypes associated with genetic deletion screens in mice from mouse genome informatics database (http://www.informatics.jax.org/). We manually curated the phenotypes which resulted in lethality during pre-natal stage or in developmental abnormalities in brain, kidney, and liver. The complete set of phenotypes and their associated genes is given in Supplementary Data S6. **e** For each CFS, we identified samples with inactivating mutations, and for each such sample, we identified 10 wildtype samples with similar expression of the CSF, computed median PSI across EP events in each sample and estimated the standard deviation across the 10 control wildtype samples. The figure shows the density plot of standard deviations across CSFs. To ensure the homogeneity of control samples in terms of their median EP splicing, we discarded all CSFs for which the control samples had a standard deviation of > 0.1. **f** Proportion of critical positive regulators (marked CSF in the plot) and non-critical (marked as nCSF in the plot) of the EP in brain, kidney, and liver bearing cancer-specific hotspot mutations (from Seiler et al., 2018). **g** Distribution of the number of CSFs that underwent upregulation (tumor vs. normal fold change (FC) > 1.5) across the patients of brain, liver and kidney cancers. **h** Boxplot distribution of the mutation rate (total number of mis-sense mutations per sample) of CSF in early and late stages of kidney (upper panel, n = 45) and liver (lower panel, n = 156) cancer. Two-sided p-values from Wilcoxon's test are shown. **i** Bar plots showing the enrichment (Odds ratio) of the EP and EN splicing events among the CSF and other splicing factors in brain, kidney, and liver. FDR corrected two-sided p-values from Fisher's test are shown. In boxplots (**c, h)**, the horizonal line in the middle is the median value and the lower and upper edges of the boxes correspond to the 25th and 75th percentiles. Extending vertically upwards/downwards of the boxes are the lines showing 1.5 times the interquartile range (i.e., distance between 25th and 75th percentile). Dots are the outliers. Source data for these figures are provided as a Source Data file.

Supplementary Figure 5

a

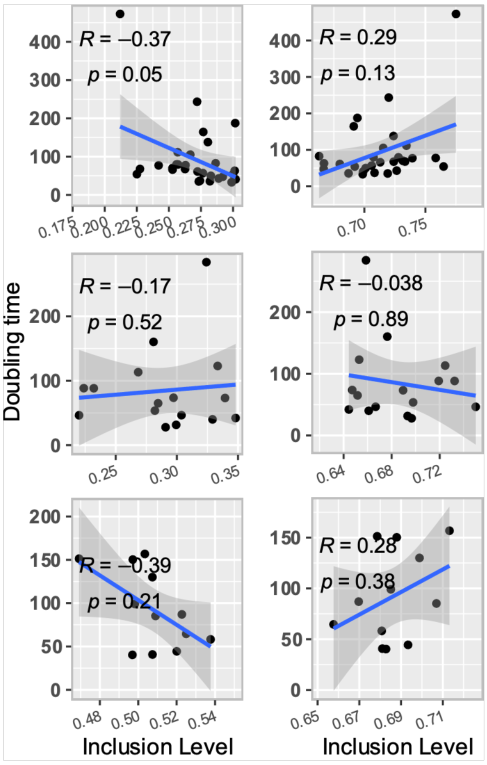

b

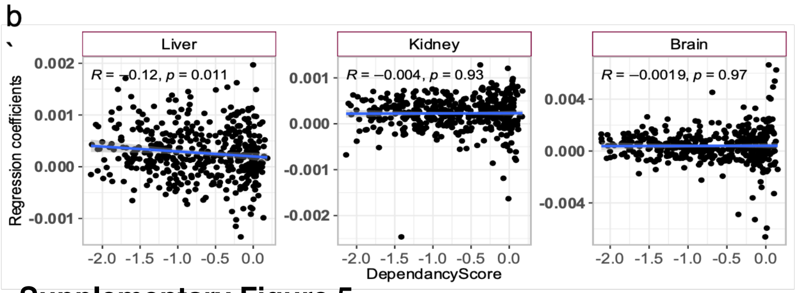

Supplementary Figure 5

a Scatter plot of median inclusion level of EP and EN events with the doubling time of cancer cell lines (obtained from DepMap portal) corresponding to brain, kidney, and liver.
Spearman's correlation and two-sided p-value are shown. Blue lines and shaded grey areas respectively depict the best fitting lines and 95% confidence intervals based on a linear regression between median EP inclusion level and doubling time of cell lines. **b** Scatter plot for the correlation of regression coefficients of CSFs with the mean dependency score of CCLE cancer cell lines from DepMap project. Pearson's correlation coefficient and two-sided p-values are shown. Each dot is a CSF; blue lines and shaded grey areas respectively depict the best fitting lines and 95% confidence intervals based on linear regression. Source data for these figures are provided as a Source Data file.

Supplementary Figure 6

a

Transmembrane-region domain

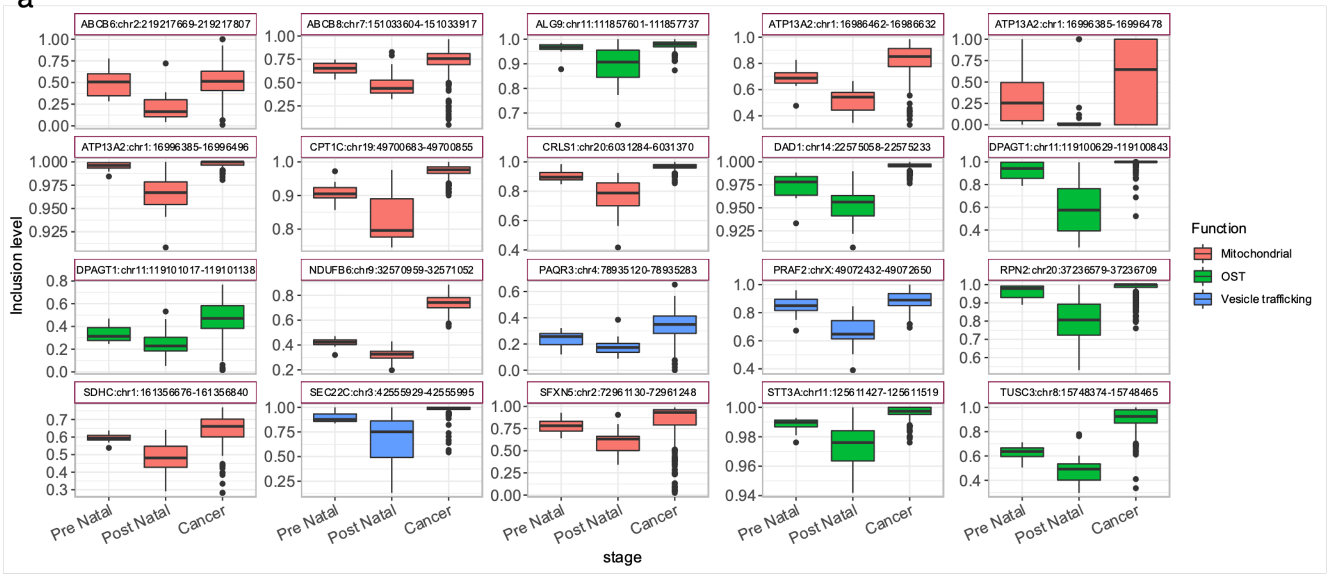

WD40- domain

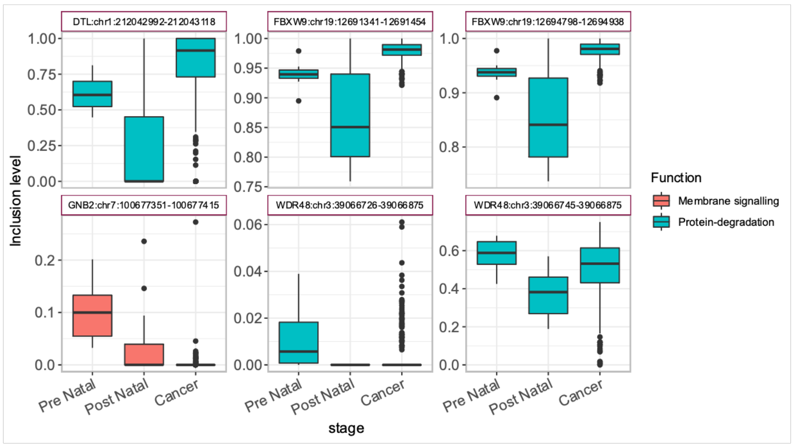

Nitrosylation domain

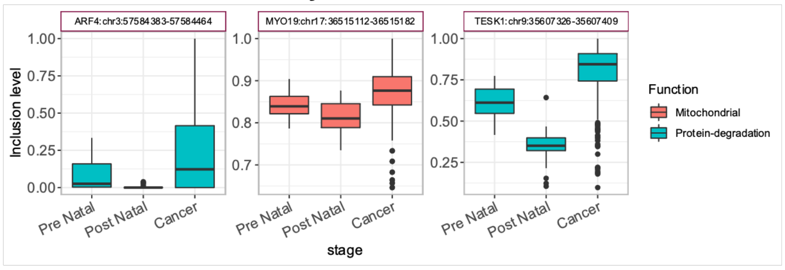

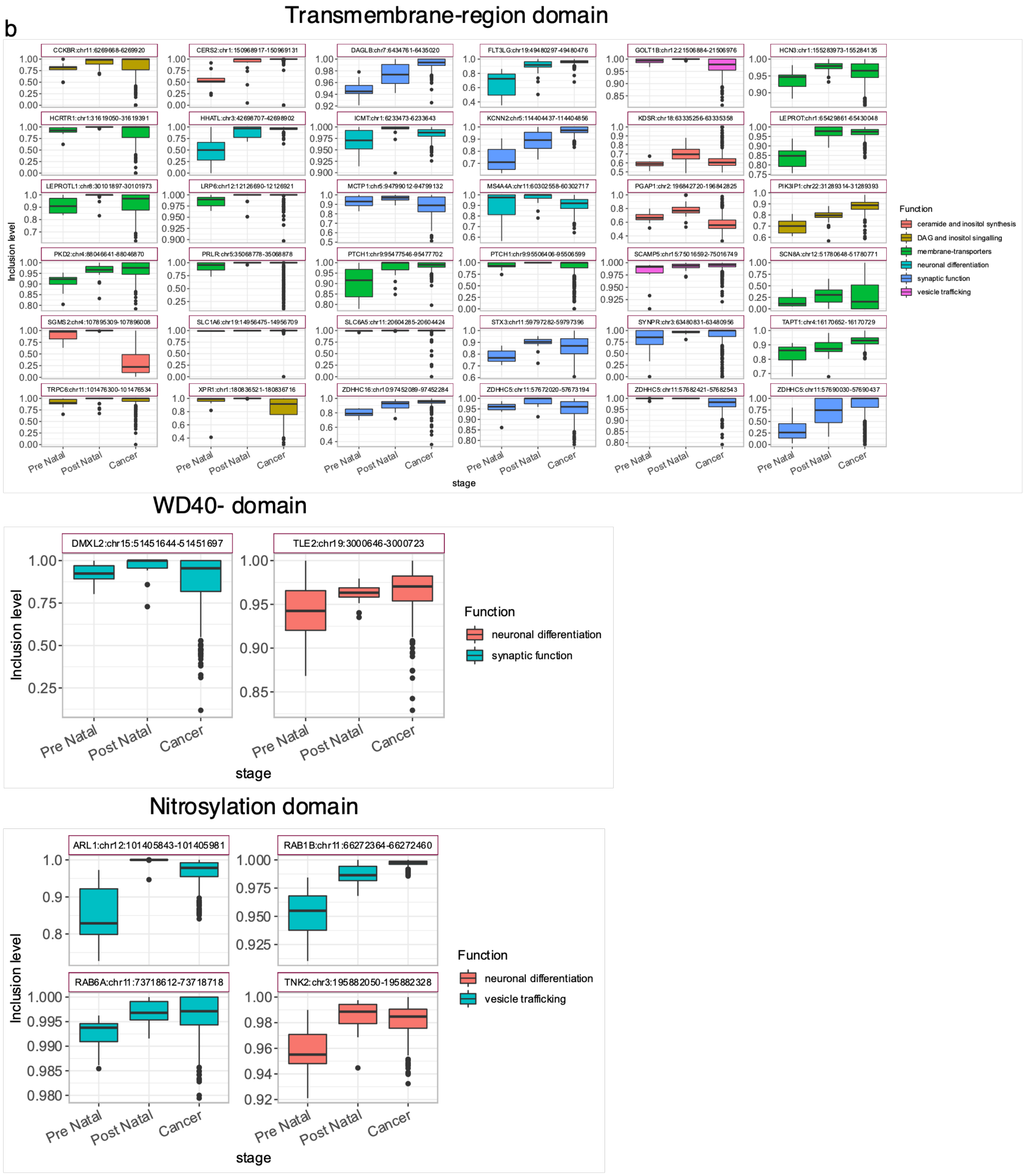

Supplementary Figure 6

Inclusion level of functionally coordinated EP (**a**) and EN (**b**) exons encoding TRD, WD40 and nitrosylation domain in pre-natal, post-natal, and cancer samples of brain. Text inside the stripes of boxplots are gene names and exon coordinates (hg38) and color represents biological functions performed by the genes. A detailed description is provided in the Discussion section. In all cases, n = 11 for pre-natal, n = 21 for post-natal, and n = 509 for cancer samples. In boxplots, the horizonal line in the middle is the median value and the lower and upper edges of the boxes correspond to the 25th and 75th percentiles. Extended vertically upwards/downwards of the boxes are the lines showing 1.5 times the interquartile range (i.e., distance between 25th and 75th percentile). Dots are the outliers. Source data for these figures are provided as a Source Data file.

Supplementary Methods

Computational drug repositioning

We performed virtual screening process using Autodock Vina [14]. The 3D structure of the FOXM1 and MYC proteins were downloaded from the RCSB-PDB [15] and further refinement was done using Open Babel software [16] and the drug library was created by downloading the molecules from the ZINC database [17] where we considered only those drug molecules which are either FDA approved or are under clinical trials. The ligand and the receptor files were prepared in the 'pdbqt' file format, and the center and the grid size of the receptor molecules was computed using UCSF Chimera software [18]. Based on the affinity score and RMSD value, we proposed some potential drug molecules.

Supplementary Table 1. List of drugs which can target *MYC* and *FOXM1*

**TF name**	**FDA approved drug**
FOXM1	Phthalocyanine
FOXM1	Bemcentinib
FOXM1	MK-3207
FOXM1	Laniquidar
FOXM1	Anatibant
MYC	Phthalocyanine
MYC	Tarazepide
MYC	Bemcentinib
MYC	Temoporfin
MYC	Gliquidone

Supplementary note 1 - Use of PEGASAS

In this investigation, we chose to use the previously proposed PEGASAS [1] approach to identify the splicing events whose PSI value co-varied with the expression of embryonic pathways (Fig. 1A/B). The rationale underlying PEGASAS is that the critical molecular entities underlying the phenotypic variations across disease, development, and ageing, co-vary with the biological processes and pathways linked with the specific phenotypic states. Therefore, the task of identifying the molecular changes associated with a phenotype is reduced to first identifying the processes and pathways linked to the phenotype and then in a subsequent step identifying molecular changes co-varying with those processes and pathways. This concept has been successfully applied to investigate alternative splicing in prostate, breast, and lung cancer datasets by Yi Xing's group [1].

While the use of differential analysis, which relies on assessing the significance of difference between two biological conditions/states (for instance embryonic vs. adult) is appealing in its directness, the functional interpretation of the resulting gene list (exons in our case) is challenging and requires additional steps of gene set analysis, which can be unstable [2,3] and heavily depends on the choice of software [4].

In contrast, in our approach, we first identify hundreds of KEGG pathways which show preferentially high activity during the pre-natal stage of the development (Fig 1B) and select the splicing events which exhibit a significant co-variation with those embryonic pathways. Such splicing events, by virtue of their preferential correlation with embryonic pathways, have a more straightforward interpretation which does not suffer from aforementioned pitfalls for their functional interpretation. To sum up, this approach enables us to identify the exons which are:

a. preferentially embryonic in nature to begin with, and importantly,
b. are correlated amongst each other, i.e., they change in coordinated fashion across developmental timepoints, which is consistent with previous publications showing the coordinated change of several hundred to thousands of exons in response to diverse biological signals and contexts [5–7]. Also, the multivariate structure of exon

inclusions has been successfully used recently to discover the sQTLs across GTEx tissues [8], further supporting our correlation based approach.

Below, we show that the PEGASAS based approach is superior in delivering these goals as compared to the conventional approach of differential splicing. For differential splicing, we used Wilcoxon test followed by FDR correction as has been performed previously [9] and called an event to be embryonic positive if ∆PSI (prenatal – postnatal PSI) values were > 0.2 with an FDR <= 0.05 in developing brain. We intersected this new set of embryonic events with our pathway-based EP events and obtained unique EP events in our approach (referred here to as pathway-only, pathway-based events that additionally qualified the differential splicing criteria above (referred to as pathway+wilcox), and the unique Wilcoxon based events (referred here to as wilcox-only).

First, we assessed the coordination in splicing within each of the three sets of exons by calculating the within-group pair-wise Pearson correlation coefficients among their inclusion level across timepoints. As shown in the boxplots below, we observed that pathway-based events (even the unique ones) are significantly more correlated with each other than the wilcox-only group.

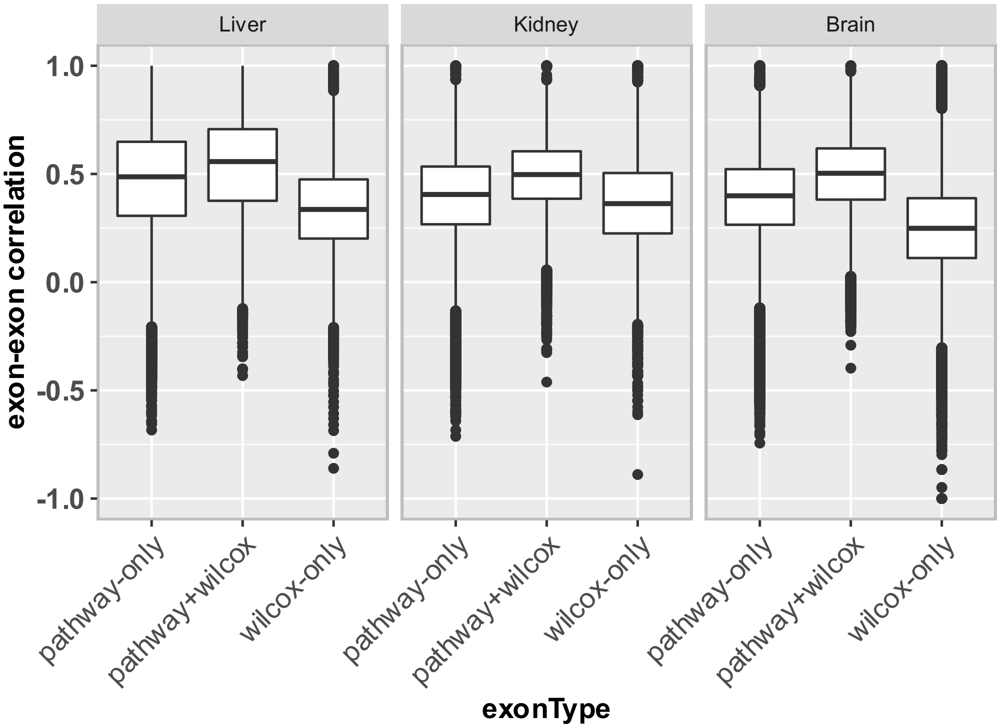

The coordinated nature of splicing events was an important aspect of our study which has been relatively neglected so far in the field of cancer biology and which helped us elucidate the important associations of the coordinated embryonic splicing events with cellular functions such as N-linked protein glycosylation and retrograde transport (Fig 3, 7 and S6).

Moreover, while the events identified by the pathway approach (as shown in Fig. 1C) and even the events unique to pathway-based approach (as shown below) were enriched for several GO terms related to the embryonic development of brain, notably, the events unique to differential events were not enriched for any functional category as shown below.

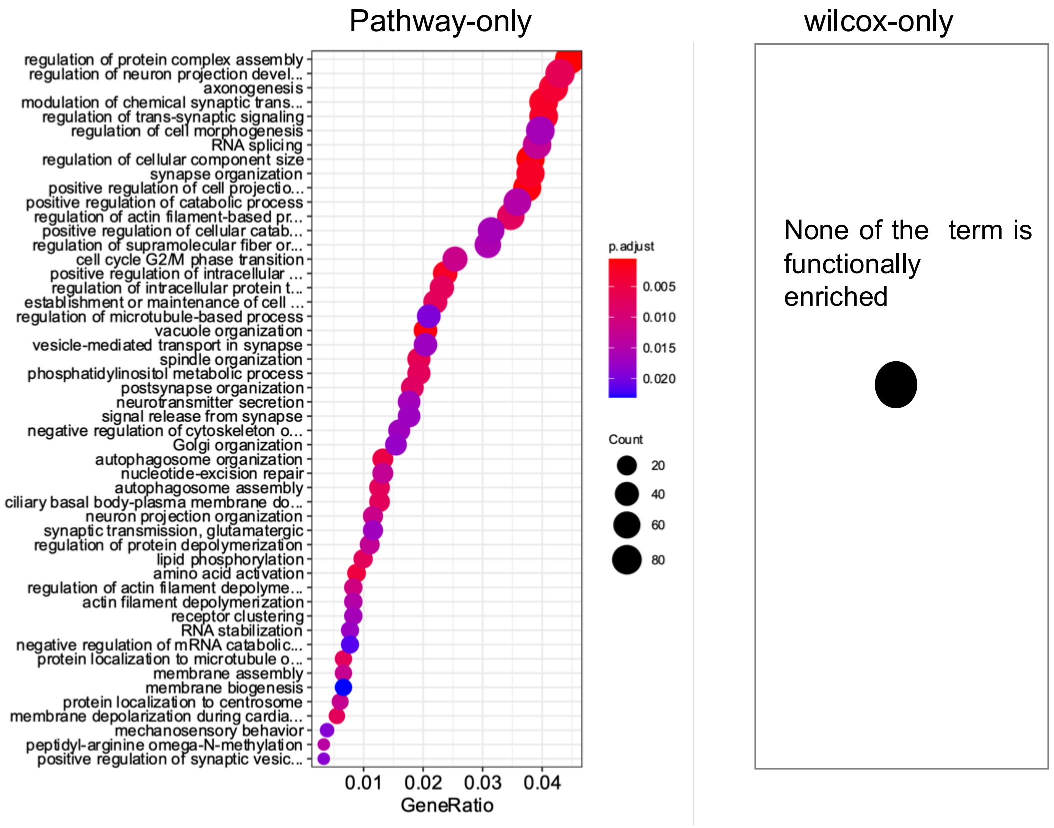

Finally, we found that the recapitulation of embryonic splicing in cancer holds up even in the pathway-only events but exhibits a much weaker trend for wilcox-only events that was statistically significant in only one of the three tissues.

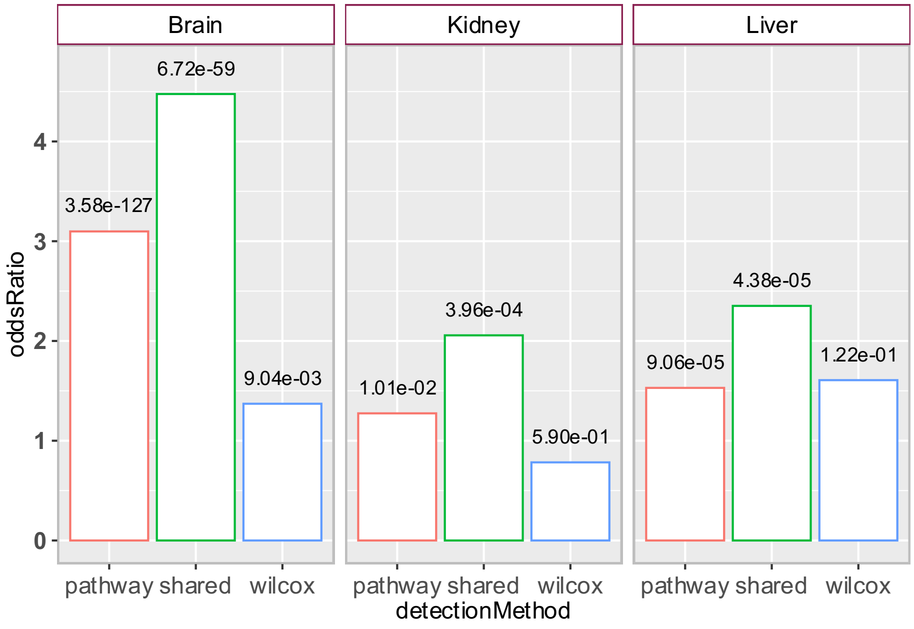

Taken together, these analyses suggest that pathway-based approach is more effective in detecting the coordinated set of context-specific events that are better recapitulated in cancer as well as provides a more direct functional interpretation of splicing events correlated.

Supplementary note 2 - Assessment of embryonic reversal of cancer splicing using pseudo-alignment free approach

To save time, computation and storage requirements in this work, we have used pre-quantified RNA-seq datasets for GTEx and TCGA cohorts from the toil-hub [10] of UCSC-xena browser as explained in the methods section. Briefly, we downloaded the transcript level TPMs datasets which were generated with pseudo-alignment based method called Kallisto, and used SUPPA2 [11] to quantify the exon skip events using this dataset. To validate the conclusion derived from 'Kallisto + SUPPA2' using an alternative pipeline not relying on pseudoalignment, we processed the controlled access transcriptomic data from GTEx (healthy brain) and TCGA (Glioblastoma) along with the developmental data of

brain using a pseudoalignment free approach using the STAR 2-pass strategy [12] to generate the genomic alignments for each of the transcriptomic datasets. We then used rMATs [13] to quantify the inclusion level of exon skip events in these three cohorts and assessed the embryonic reversal of cancer splicing. Off note, we observed a high across sample correlation between the inclusion level estimates derived from these two pipelines (median correlation coefficient 0.84).

Furthermore, as shown in the scatter plot below, we observed that the cancer associated changes in the inclusion level of exon skip events (i.e., TCGA – GTEx ΔPSI) was significantly positively correlated with development associated changes (i.e., Prenatal – Postnatal ΔPSI).

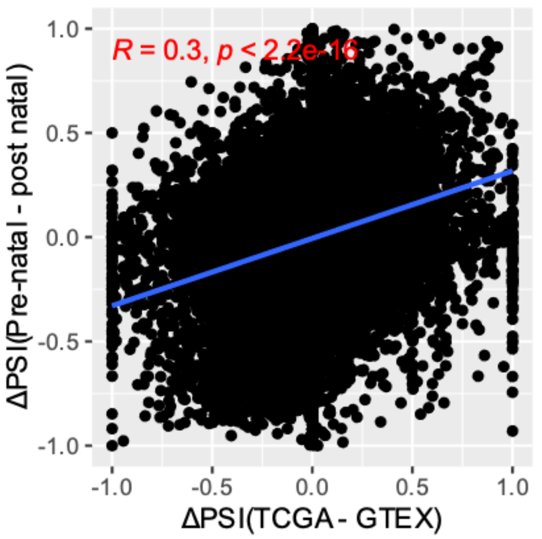

Further, using this new splicing data generated with rMATs, we defined a new set of embryonic splicing events (namely, EP for embryonic positive and EN for embryonic negative) by using Wilcoxon's rank sum test (absolute median inclusion difference > 0.2 and FDR < 0.1) between pre-natal and post-natal inclusion level of the exon skip events. We compared this set of the embryonic splicing events, using Fisher's exact test, with the cancer specific events (namely 'Increased' for events with increased inclusion and 'decreased' for events with decreased inclusion in TCGA cancer samples as compared to GTEx normal samples) which were derived by using a similar approach. We observed

a very strong and significant enrichment of the EP events among the events increased in cancer (and vice-versa for EN events), as shown in the bar-plots below.

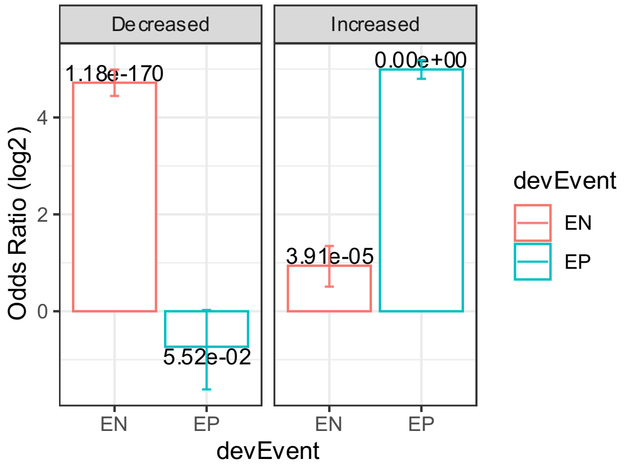

These results indicate that conclusions generated using our Kallisto + SUPPA2 pipeline are statistically robust and can be re-capitulated using independent data processing methods.

Supplementary references

1. Phillips, J. W. *et al.* Pathway-guided analysis identifies Myc-dependent alternative pre-mRNA splicing in aggressive prostate cancers. *Proc. Natl. Acad. Sci.* **117**, 5269–5279 (2020).
2. Gaudet, P. & Dessimoz, C. Gene ontology: Pitfalls, biases, and remedies. in *Methods in Molecular Biology* vol. 1446 (2017).
3. Jacobson, M., Sedeño-Cortés, A. E. & Pavlidis, P. Monitoring changes in the Gene Ontology and their impact on genomic data analysis. *Gigascience* **7**, (2018).
4. Xie, C., Jauhari, S. & Mora, A. Popularity and performance of bioinformatics software: the case of gene set analysis. *BMC Bioinformatics* **22**, (2021).
5. Bland, C. S. *et al.* Global regulation of alternative splicing during myogenic differentiation. *Nucleic Acids Res.* **38**, (2010).
6. Warzecha, C. C. *et al.* An ESRP-regulated splicing programme is abrogated during the epithelial-mesenchymal transition. *EMBO J.* **29**, (2010).
7. Moore, M. J., Wang, Q., Kennedy, C. J. & Silver, P. A. An alternative splicing network links cell-cycle control to apoptosis. *Cell* **142**, (2010).
8. Garrido-Martín, D., Borsari, B., Calvo, M., Reverter, F. & Guigó, R. Identification and analysis of splicing quantitative trait loci across multiple tissues in the human genome. *Nat. Commun.* **12**, (2021).
9. Zhang, Y. *et al.* Pan-cancer analysis of clinical relevance of alternative splicing events in 31 human cancers. *Oncogene* **38**, 6678–6695 (2019).
10. Vivian, J. *et al.* Toil enables reproducible, open source, big biomedical data analyses. *Nature Biotechnology* vol. 35 (2017).
11. Trincado, J. L. *et al.* SUPPA2: Fast, accurate, and uncertainty-aware differential splicing analysis across multiple conditions. *Genome Biol.* **19**, (2018).
12. Dobin, A. *et al.* STAR: Ultrafast universal RNA-seq aligner. *Bioinformatics* **29**, (2013).
13. Shen, S. *et al.* rMATS: Robust and flexible detection of differential alternative splicing from replicate RNA-Seq

data. *Proc. Natl. Acad. Sci. U. S. A.* **111**, (2014).
14. Trott, O. & Olson, A. J. AutoDock Vina: Improving the speed and accuracy of docking with a new scoring function, efficient optimization, and multithreading. *J. Comput. Chem.* (2009) doi:10.1002/jcc.21334.
15. Berman, H. M. *et al.* The Protein Data Bank. *Nucleic Acids Research* vol. 28 (2000).
16. O'Boyle, N. M. *et al.* Open Babel: An Open chemical toolbox. *J. Cheminform.* **3**, (2011).
17. Irwin, J. J. & Shoichet, B. K. ZINC - A free database of commercially available compounds for virtual screening. *J. Chem. Inf. Model.* **45**, (2005).
18. Pettersen, E. F. *et al.* UCSF Chimera - A visualization system for exploratory research and analysis. *J. Comput. Chem.* **25**, (2004).
