# Supplementary material for: Broad misappropriation of developmental splicing profile by cancer in multiple organs
Source: Nat Commun. 2022 Dec 12;13:7664. doi: 10.1038/s41467-022-35322-1 (PMC9744839; doi:10.1038/s41467-022-35322-1)
Supplement: Supplementary file 3 — Description of Additional Supplementary Files [file 41467_2022_35322_MOESM3_ESM.pdf]

Description of Additional Supplementary Files

File Name: Supplementary Data 1

Description: List of pre-natal and post-natal developmental timepoints used in this study for brain, kidney, and liver.

File Name: Supplementary Data 2

Description: List of GO terms enriched among the constituent genes of embryonic pathways in brain, kidney, and liver.

File Name: Supplementary Data 3

Description: List of EP and EN splicing events identified in brain, kidney, and liver.

File Name: Supplementary Data 4

Description: List of GO terms enriched among the host genes of EP and EN exons.

File Name: Supplementary Data 5

Description: Regression coefficients of the splicing factors corresponding to the first component of the PLSR model for embryonic splicing.

File Name: Supplementary Data 6

Description: List of manually curated human phenotype ontology terms which resulted in lethality during pre-natal stage or in developmental abnormalities in brain, kidney, and liver.

File Name: Supplementary Data 7

Description: List EP and EN exons containing transmembrane-regions, WD40 or nitrosylation domain in brain, liver, and kidney, along with their host gene names and rank of exon in the major transcript.

File Name: Supplementary Data 8

Description: Examples of EP and EN exons containing transmembrane-region, WD40 or nitrosylation domain which are previously reported to be alternatively spliced using experimental investigations in various human diseases.
