# Supplementary material for: Broad misappropriation of developmental splicing profile by cancer in multiple organs
Source: Nat Commun. 2022 Dec 12;13:7664. doi: 10.1038/s41467-022-35322-1 (PMC9744839; doi:10.1038/s41467-022-35322-1)
Supplement: Supplementary file 12 — Reporting Summary [file 41467_2022_35322_MOESM12_ESM.pdf]

nature portfolio

Corresponding author(s): Arashdeep Singh, Sridhar Hannenhalli

Last updated by author(s): 2022/11/29

Reporting Summary

Nature Portfolio wishes to improve the reproducibility of the work that we publish. This form provides structure for consistency and transparency in reporting. For further information on Nature Portfolio policies, see our Editorial Policies and the Editorial Policy Checklist.

Statistics

For all statistical analyses, confirm that the following items are present in the figure legend, table legend, main text, or Methods section.

n/a	Confirmed	
☐	☒	The exact sample size (*n*) for each experimental group/condition, given as a discrete number and unit of measurement
☐	☒	A statement on whether measurements were taken from distinct samples or whether the same sample was measured repeatedly
☐	☒	The statistical test(s) used AND whether they are one- or two-sided<br>*Only common tests should be described solely by name; describe more complex techniques in the Methods section.*
☐	☒	A description of all covariates tested
☐	☒	A description of any assumptions or corrections, such as tests of normality and adjustment for multiple comparisons
☐	☒	A full description of the statistical parameters including central tendency (e.g. means) or other basic estimates (e.g. regression coefficient) AND variation (e.g. standard deviation) or associated estimates of uncertainty (e.g. confidence intervals)
☐	☒	For null hypothesis testing, the test statistic (e.g. *F*, *t*, *r*) with confidence intervals, effect sizes, degrees of freedom and *P* value noted<br>*Give P values as exact values whenever suitable.*
☒	☐	For Bayesian analysis, information on the choice of priors and Markov chain Monte Carlo settings
☒	☐	For hierarchical and complex designs, identification of the appropriate level for tests and full reporting of outcomes
☐	☒	Estimates of effect sizes (e.g. Cohen's *d*, Pearson's *r*), indicating how they were calculated

Our web collection on statistics for biologists contains articles on many of the points above.

Software and code

Policy information about availability of computer code

Data collection	The codes used to download raw/processed data are deposited on github (https://github.com/hannenhalli-lab/AltSplDevCancer) and the corresponding DOI is as follows: 10.5281/zenodo.7325464
Data analysis	The codes used for data analysis is deposited on github (https://github.com/hannenhalli-lab/AltSplDevCancer/)<br>Softwares used for data analysis are as follows<br>Kallisto: v0.46.1<br>SUPPA2: v2.3<br>maftools: 2.2.10

For manuscripts utilizing custom algorithms or software that are central to the research but not yet described in published literature, software must be made available to editors and reviewers. We strongly encourage code deposition in a community repository (e.g. GitHub). See the Nature Portfolio guidelines for submitting code & software for further information.

Data

Policy information about availability of data

All manuscripts must include a data availability statement. This statement should provide the following information, where applicable:

- Accession codes, unique identifiers, or web links for publicly available datasets
- A description of any restrictions on data availability
- For clinical datasets or third party data, please ensure that the statement adheres to our policy

The public RNA-seq datasets for human cancers were generated by TCGA consortium (https://www.cancer.gov/tcga) and are publicly available from the 'toilhub' of UCSC-Xena browser [98] (UCSC-Xena-TCGA). The public RNA-seq datasets for healthy human tissues were generated by GTEx consortium and publicly available from

the 'toilhub' of the UCSC-Xena browser [98] (UCSC-Xena-GTEx). The public mutation calls and copy number amplifications from whole exome sequencing data of human cancers are publicly available from TCGA genomics data commons portal (https://portal.gdc.cancer.gov/) [119]. The public RNA-seq datasets spanning multiple stages during human organogenesis are publicly available and downloaded from array express (E-MTAB-6814) [29]. The public clinical and survival data of cancer patients is publicly available and downloaded from Pan-Cancer Atlas initiative (TCGA-clinical) [120]. The public mapping of PFAM domains to hg38 assembly was performed by a previous study and the mapping coordinates are publicly available to download from the prot2hg database (http://www.prot2hg.com) [106]. The public data for frequently mutated splicing factors with a significant evidence for their cancer driver gene activity is publicly available and downloaded from the Table S1 of Seiler et al. [54]. The public RNA-seq datasets for shRNA knockdown of splicing factors and corresponding controls for HepG2 cell line were downloaded from ENCODE database (ENCODE-shRNA-HepG2) [48]. The public data for doubling time, RNA-seq, and genome-wide dependency score for cancer cell lines are publicly available and downloaded from the DepMap portal release 22Q2 (DepMaP) [121]. The public single cell RNA-seq data for glioblastoma patients is publicly available and downloaded from the Single Cell Portal of the Broad Institute under the accession code SCP393 (sc-GBM) [113]. The public single cell RNA-seq data for healthy brain samples is publicly available and downloaded from Allen Brain Atlas (sc-Brain) [114]. The public single cell RNA-seq data for liver cancer is publicly available and downloaded from GEO database under the accession code GSE125449 (sc-LIHC) [115]. Single cell RNA-seq data for healthy liver is publicly available and imported with HumanLiver package in R (sc-Liver) [116]. The public data for differentially expressed genes following the deletion of TFs across multiple cell lines is publicly available and downloaded from KnockTF database (KnockTF) [56]. The public ChIP-seq datasets for the genome-wide binding of TFs across multiple model systems is publicly available and downloaded from the GitHub repository of the TFEA.ChIP library in R 55 (TFEA.ChIP). The public data for human phenotype ontology terms is publicly available and downloaded from The Jackson laboratory (HPO) [122]. Gene and transcript coordinates for hg38 assembly were downloaded from Gencode (Gencode V23) [123]. The remaining data generated in this study are provided with this paper as supplementary files and source data file.

Field-specific reporting

Please select the one below that is the best fit for your research. If you are not sure, read the appropriate sections before making your selection.

[x] Life sciences [ ] Behavioural & social sciences [ ] Ecological, evolutionary & environmental sciences

For a reference copy of the document with all sections, see nature.com/documents/nr-reporting-summary-flat.pdf

Life sciences study design

All studies must disclose on these points even when the disclosure is negative.

Sample size	No sample size calculations were performed in this study. This study used multiple publicly available datasets from TCGA, GTEx, ENCODE, and an independent study (Cardoso-Moreira et al. 2019). The sample size for TCGA/ GTEx related analysis was based on number of patients/ healthy individuals analyzed by these consortia. In the developmental RNA-seq data from Brain, Liver, and Kidney (Cardoso-Moreira et al., 2019), the developmental time-points were chosen to sample the pre-natal development as early as possible and to cover the most important stages in organ development as well as aging. In the case of shRNA knockdown data of splicing factors from performed by ENCODE consortrium, experiments were performed in biological duplicates.
Data exclusions	No samples from TCGA, GTEx or Cardoso-Moreira et al. (2019) were excluded from our anaysis. For shRNA knockdown data from ENCODE, we only considered the cases where shRNA knockdown resulted in atleast 50% reduction in the expression of the target gene. We also exlucded the control set of experiments with high inter-replicate variablity (i.e., the cases where Pearson's correlation coefficient between two replicates < 0.90).
Replication	Publicly available shRNA knockdown data in HepG2 from ENCODE cell line consisted of two biological replicates for each splicing factors. In Cardoso-Moreira et al. (2019), the developmental transcriptome consisted 1-4 biological replicates across timepoints in Brain, Liver, and Kidney. In their investigation, Cardoso-Moreira et al. (2019) excluded the libraries caused by low RNA quality and the libraries that showed a Spearman's correlation coefficient with its biological replicates lower than 0.9.
Randomization	No data randomizations were applicable/performed in this study. However, for the embryonic reversal of cancer splicing, we performed a random sampling of all the alternatively spliced exons to show that a random set of exons are not likely to be over-represented in cancer.
Blinding	Blinding was not applicable as no direct experimental observations were made which could be subjected to human bias.

Reporting for specific materials, systems and methods

We require information from authors about some types of materials, experimental systems and methods used in many studies. Here, indicate whether each material, system or method listed is relevant to your study. If you are not sure if a list item applies to your research, read the appropriate section before selecting a response.

Materials & experimental systems

n/a	Involved in the study
☒	☐ Antibodies
☒	☐ Eukaryotic cell lines
☒	☐ Palaeontology and archaeology
☒	☐ Animals and other organisms
☒	☐ Human research participants
☒	☐ Clinical data
☒	☐ Dual use research of concern

Methods

n/a	Involved in the study
☒	☐ ChIP-seq
☒	☐ Flow cytometry
☒	☐ MRI-based neuroimaging
